# Effect of an ICU diary on psychiatric disorders, quality of life, and sleep quality among adult cardiac surgical ICU survivors: a randomized controlled trial

**DOI:** 10.1186/s13054-020-2797-7

**Published:** 2020-03-06

**Authors:** Shuo Wang, Hui-Ning Xin, Chiang Chung Lim Vico, Jin-Hua Liao, Sai-Lan Li, Na-Mei Xie, Rong-Fang Hu

**Affiliations:** 1 School of Nursing, Fujian Medical University, 1 Xue Yuan Road, University Town, Fuzhou, 350122 China; 2 Fujian Provincial Hospital, Fuzhou, China; 3 School of Nursing, The Hong Kong Polytechnic University, Kowloon, Hong Kong; 4 Fujian Medical University Union Hospital, Fuzhou, China

**Keywords:** Intensive care unit, Dairies, Post-traumatic stress disorder, Quality of life, ICU memory

## Abstract

**Background:**

Although studies on the effectiveness of the use of ICU diaries on psychiatric disorders and quality of life have been published, the results still seem to be controversial. The study aimed to determine the effects of using an ICU diary on psychiatric disorders, sleep quality, and quality of life (QoL) in adult ICU survivors in China.

**Methods:**

One hundred and twenty-six patients who underwent a scheduled cardiac surgery and were expected to stay ≥ 24 h in ICU were randomized to two groups (63 in each group). The patients in the intervention group received the use of ICU diaries during the period of post-ICU follow-up, while the patients in the control group received usual care without ICU diaries. The primary outcome was significant PTSD symptoms (Chinese version of Impact of Event Scale-Revised, IES-R; total score ≥ 35 was defined as significant PTSD symptoms) and its severity in patients 3 months post-ICU. The secondary outcomes included memories of the ICU at 1 month, QoL (Medical Outcomes Study 36-item Short-Form, SF-36), sleep quality (Pittsburgh Sleep Quality Index Questionnaire, PSQI), anxiety, and depression symptoms (Hospital Anxiety and Depression Scale, HADS) at 3 months.

**Results:**

Eighty-five and 83 patients completed the follow-up interviews at 1 month and 3 months post-ICU, respectively. Significant PTSD symptoms were reported by 6 of 41 (14.63%) in the intervention group vs 9 of 42 (21.43%) in the control group (risk difference, − 9% [95% CI, − 2% to 21%], *P* = 0.10). There was no significant differences between groups in IES-R score, symptoms of intrusion, symptoms of avoidance, numbers of memories of feeling and delusional memories, SF-36 score and anxiety score (*P* > 0.05), while significant differences were found in symptom of hyperarousal score, numbers of factual memories and PSQI score (*P* < 0.05). No adverse effect was reported.

**Conclusions:**

Using an ICU diary is not useful for preventing PTSD symptoms and anxiety symptoms and preserving the quality of life of the patients at 3 months post-ICU, while it significantly improves the survivor’s factual memory of ICU and sleep quality, and prevents the hyperarousal symptom.

**Trial registration:**

Chinese Clinical Trial Registry, ChiCTR-IOR-16009109, registered on 28 August 2016

## Key messages

Using an ICU diary is not useful for preventing PTSD symptoms and anxiety symptoms and preserving the health-related quality of life in the patients after 3 months of discharge from ICU.

Using an ICU diary is useful for increasing the survivor’s factual memories of ICU at 1 month post-ICU, improving sleep quality, and preventing hyperarousal symptoms at 3 months post-ICU.

## Background

With the decrease of mortality in critical care in recent decades [1], concerns are growing about long-term outcomes and quality of life in the intensive care unit (ICU) survivors. In particular, more attention has been given to psychiatric disorders such as symptoms of anxiety, depression, and post-traumatic stress disorder (PTSD). It is known that patients frequently experience memory gaps and unpleasant recall after ICU discharge [2], which are often associated with the development of mental health disorders that impact negatively on the health-related quality of life in ICU survivors [3–5].

A large proportion of patients suffered from PTSD symptoms, with rates ranging from 15.93 to 25.69% in the first year following discharge from the ICU [6]. Given the high prevalence of these psychiatric disorders and their potential negative impacts on long-term quality of life in ICU survivors, effective interventions to preventing PTSD and promoting mental health recovery in ICU survivors are urgently needed.

Since the beginning of the year 2000 [7], the use of ICU diaries has been used as a tool to help the patients recover physically and mentally after ICU admission [7–14]. ICU diaries filled in by ICU staff and/or family could provide patients with a factual recall of their ICU experience, which may help to fill in the memory gap and enable reconstruction of their stories of ICU experience [15]. Reading ICU diary may be a process of repetition and reinforcement of factual memory, which enables patients to distinguish factual events from hallucinations and delusions.

Although studies on the effectiveness of the use of ICU diaries on psychiatric disorders and quality of life have been published, the results still seem to be controversial. Several trials indicated that ICU diaries reduced PTSD symptoms [7, 8, 16], but results by meta-analysis showed that ICU diaries improved quality of life, decreased symptoms of anxiety and depression but not PTSD [17, 18]. An assessor-blinded, multicenter RCT in 35 French ICUs did not support ICU diaries for preventing symptoms of PTSD, anxiety, and depression [19]. A diary and a psychoeducation program reduced anxiety, depression, and PTSD symptoms 3 months after ICU discharge [7]. In a non-randomized trial, keeping an ICU diary with photos improved health-related QoL during the 3-year follow-up period after ICU [14]. Evidences of the usefulness of ICU diary seem to lack harmony. Many factors may contribute to the differences across these studies: the time of outcome assessment, instrument used, population studied, cultural context, difference of ICU diary intervention, and so on. Most research in this area has focused on general ICU or medical ICU settings and has been conducted in western countries and Europe. Few studies of ICU diary conducted in the patients undergoing cardiac surgery and intensive care unit therapy. Moreover, evidence from China is limited.

In addition, sleep disturbance is another common problem for ICU survivors including insomnia, nightmares, and poor-quality sleep [20]. Psychiatric symptoms, such as trauma-related symptoms and depressive symptoms, were associated with a higher likelihood of post-ICU sleep disturbances [21]. Sleep is crucial for rest, recovery, and well-being. No studies have yet evaluated the effects of using an ICU diary on the sleep of ICU survivors. This study was designed to examine the effects of ICU diaries on psychological outcomes, quality of life, and sleep quality among adult cardiac surgical ICU survivors in a Chinese context.

## Methods

### Study design

This study was a prospective randomized controlled parallel-group clinical trial performed within two cardiac surgery ICUs (CSICU) of two university hospitals in Fuzhou, China. It was approved by the Hospital and Fujian Medical University Research Ethics Boards. The trial was registered in the Chinese Clinical Trials Registry (ChiCTR-IOR-16009109). Written informed consent for participation in the study was obtained before surgery.

### Participants and study settings

Study participants were recruited from February 2016 to January 2017. The inclusion criteria were (1) underwent elective cardiac surgery; (2) age ≥ 18 years; (3) length of ICU stay ≥ 24 h; (4) ability to communicate verbally and understand the questionnaires administered before surgery and after being transferred from the ICU. Exclusion criteria were (1) presence of cranio-cerebral diseases; (2) history of craniotomy, severe cognitive impairments, or neurological diseases; (3) postoperative unconsciousness or coma; (4) length of ICU stay ≥ 10 days. Reasons for study termination criteria were (1) patient request for withdrawal; (2) re-admitted to the hospital or ICU; (3) lost to follow-up; (4) death within 3 months post-ICU; (5) serious adverse reactions. Staff members were asked to continue all routine postoperative medical care such as management of mechanical ventilation during the study. ICU nurses had been trained by the researchers to fill in the diary at the beginning of the study. The nurses in charge of the patient would contribute to the diary if a patient was assigned to the intervention group.

### Intervention and randomization

A random sequence of 126 numbers was generated using a web-based random number generator (http://tools.medsci.cn/rand) and sorted from small to large by a third-party researcher who was blind to the patients and did not participate in the clinical trial. The first 63 numbers were the intervention group, and the last 63 numbers were the control group. The allocation was concealed using the closed-envelope method and kept by the third researcher. After randomization, an ICU diary was filled in by the researchers and the ICU nurses in the intervention group during the postoperative ICU stay. The diary was detailed to the patient in the general ward at 1 week after discharge from ICU and reviewed at 1 month post-ICU by phone. The control group received usual ICU care and usual ward visit at 1 week and follow-up interview by phone at 1 month post-ICU while no ICU diary.

### ICU diary writing

Based on the Whiston Hospital Critical Care Unit’s patient diary guidelines [22] and methods reported by other researchers [15, 16], an ICU diary outline was designed by the researchers in this study. The frame of diary was standardized with the following headings: ward events (the time points of turning lights on and off, alarm noises, etc.); treatments (when and who performed what kind of nursing care or therapy); and details about visitors (the name of visitors and the content of communication). Photos were taken from the patient’s perspective, with a camera placed at the head of the patient’s bed, to take pictures of what the patient can see when he/she lies in the bed. Annotation of the photos and the note below the pictures helped the patient understand the ICU environment around him/her. Photos of the monitoring and treatment equipment they used were taken and were posted in their diary. The diary was initiated when the patient was admitted with an expectation of ICU stay ≥ 24 h, and ended on the last day of discharge from ICU. During the intensive care period, the diary was hanged at the patient’s bed. ICU nurses had been trained by the researchers to fill in the diary at the beginning of this study.

### Ward visit post-ICU

The ICU diary was detailed to the patient in the intervention group at 1 week after discharge from ICU to general wards. One of the researchers interviewed patients in the general wards and introduced the diaries to the patients. Before reading the diaries, the patient’s memories of the ICU stay were assessed using ICU Memory (ICUM) tool [23]. For some patients, they had delusional memories of ICU; hence, the researchers would tell them the real events and explain possible reasons for vivid memory of unreal events. The researcher explained the details of the diary and described in detail about treatments the patients had received during ICU stay. The patients were encouraged to tell out their experiences and feelings. The diaries were kept by the patients after ward visits. They were suggested to read the diaries after discharge from the hospital and share their ICU experiences with relatives and friends.

### Follow-up interview

At 1 month post-ICU, a follow-up interview was made by phone. The ICU diary was reviewed and discussed again between the researcher and the patient in the intervention group.

### Outcomes measurements

#### Primary outcome

The primary outcome was a significant PTSD symptom and its severity in patients at 3 months post-ICU as assessed by the Chinese version of Impact of Events Scale-Revised questionnaire (IES-R) [24]. The IES-R is a 22-item, 5-point Likert questionnaire of a self-report screening tool for PTSD that assesses symptoms of intrusion, avoidance, and hyperarousal (score range, 0–88). The tool was translated into Chinese and validated with high levels of sensitivity, specificity, and efficiency at the cut-off total score of 35 for significant PTSD symptoms [25], namely, that total score greater than 35 was defined as significant PTSD symptoms.

### Secondary outcomes

#### Memories of the ICU stay

Memory of ICU was assessed using the ICUM tool [23] 1 week post-ICU through a face-to-face interview in the ward (as baseline data), and at 1 month post-ICU by a phone interview (as post-intervention data). The ICUM tool was translated into Chinese and has been validated in China with high reliability and validity [26]. The tool consists of a 21-item checklist of memories of the ICU stay in 3 specific dimensions, namely, factual memories, memories of feelings, and delusional memories.

#### Health-related quality of life (QoL)

Health-related quality of life was assessed before surgery through a face-to-face interview (as baseline data) and at 3 months post-ICU by a phone interview (as post-intervention data). Health-related QoL was assessed using the Chinese version of Medical Outcomes Study 36-item Short-Form (SF-36) [27]. SF-36 generates information about 8 specific dimensions and 2 summary measures. The scores for each subscale ranged from “0” to “100,” with higher scores indicating better QoL.

#### Anxiety and depression

Anxiety and depression symptoms in patients were evaluated before the surgery and 3 months after ICU discharge using the Chinese version of Hospital Anxiety and Depression Scale (HADS) [28]. Higher scores indicate worse symptoms (score range for each subscale, 0–21).

#### Sleep quality

The patients filled out the Chinese version of the Pittsburgh Sleep Quality Index Questionnaire (PSQI) [29] to evaluate the quality of sleep before surgery and 3 months post-ICU. Higher scores indicate poorer sleep quality (0 = best sleep, 21 = worst sleep).

#### Adverse effects

Patients were asked to evaluate uncomfortable feelings and psychological or physiological reactions due to reading the diary, such as anxious, frightened, headache, crying, cataplexy, and so on during the telephone interview 1 month and 3 months after discharge from ICU by the researchers.

#### Statistical analysis and sample size

Data were analyzed using SPSS version 20.0 (IBM Corp. Armonk, NY, USA). The post hoc modified intention-to-treat (mITT) analysis and per-protocol (PP) analysis were both conducted to examine the effects. The patients in the intervention group who did not receive interventions from the randomized population were excluded to form the mITT population. Missing measurement data were replaced by mean. Missing count data were replaced by the best and worst results. Those who completed intervention and assessment were the PP population. Independent *t* test or non-parametric Mann-Whitney *U* test was used for comparison of the groups, while the chi-squared (*χ*^2^) test was used for nominal data. The differences in percentages for significant PTSD symptoms were computed with exact binomial CIs. Analysis of outcomes was performed by the third researcher who was blinded to group assignment.

The sample size was calculated based on a previous study on ICU diary [9], which found that the estimated standard derivations of mean PTSD symptoms score in ICU patients were 12.2 in the intervention group and 15.1 in the control group, and the mean difference was 8.8. It was estimated that the ICU diary intervention in this study could reduce PTSD symptoms by inducing an 8.8-point difference in total scores of IES-R between groups at 3 months post-ICU. With an effect size of 0.8 and a *P* value of 0.05, the required sample size for each group was calculated as 51 per group. With an estimate of 20% attrition, 63 per group were recruited.

## Results

### Recruitment and attrition

Among 1242 patients who were admitted to CSICU during the study period, a total of 131 patients were eligible. Finally, 126 patients were recruited and randomly allocated into 2 groups (63 in each group). In the intervention group, a total of 17 patients were withdrawn due to re-admission to the ICU/hospital (*n* = 6) and refusal to continue participating in the study (*n* = 11). In the control group, a total of 11 patients were withdrawn due to re-admission to the ICU/hospital (*n* = 3) and refusing to continue participating in the study (*n* = 11). Thus, mITT analysis was performed for 46 cases in the intervention group and 49 cases in the control group.

**Fig. 1:**
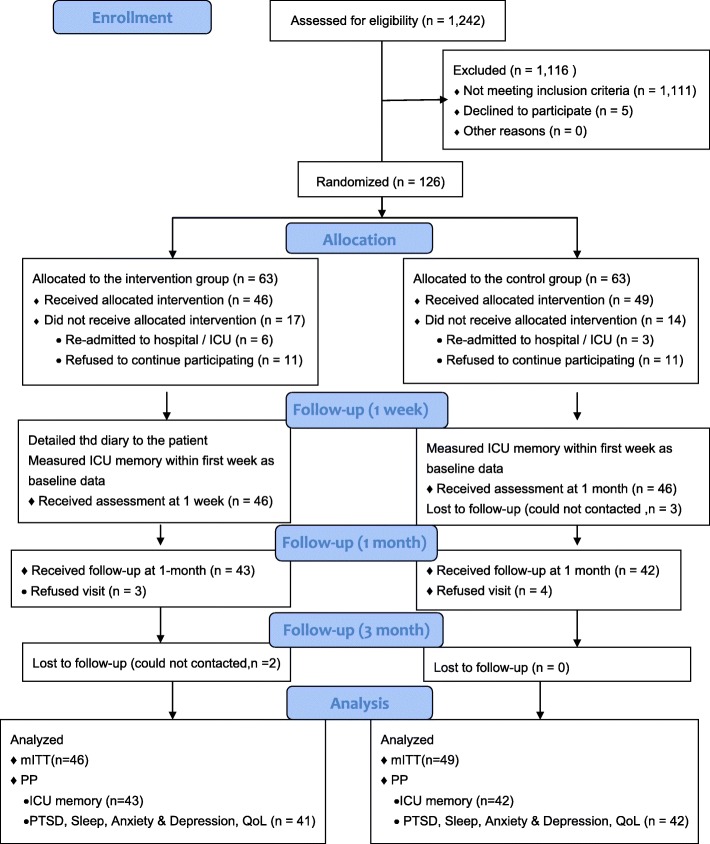
CONSORT flow diagram of the study

At 1 month post-ICU, 3 patients in the intervention group and 7 patients in the control group were lost to follow-up. Thus, PP analysis of ICU memory was conducted among 43 cases in the intervention group and 42 cases in the control group. At 3 months post-ICU, 2 patients in the intervention group were lost to follow-up. Thus, PP analysis of PTSD symptoms and QoL were conducted among 41 cases in the intervention group and 42 cases in the control group (Fig. 1).

### Participant characteristics

The findings of patients’ demographic analysis are shown in Table 1. Both study groups were comparable at baseline, with no significant differences in age, gender, height, weight, preoperative heart function, education, marital status, occupation, payment with insurance, diagnose, duration of mechanical ventilation, length of ICU stay, and duration of use of corticosteroid, sedatives, or analgesics (*P* > 0.05). But 31 patients who were withdrawn during the study due to re-admission to the ICU/hospital (*n* = 9) and/or refusal to continue the study (*n* = 22) had longer length of ICU stay, more use of glucocorticoid, sedatives, and analgesics; and longer length of mechanical ventilation than those of patients who completed the study (*P* < 0.05) (Table 2).

**Table 1:** Patient demographic characteristics at baseline

Variables	Intervention (*n* = 46)	Control (*n* = 49)	*T*/휒^2^/*Z*	*P*
Gender				
Male	29 (63%)	30 (61.2%)	0.03	0.86
Female	17 (37%)	19 (38.8%)		
Age (years), mean ± SD	53.04 ± 9.53	55.61 ± 10.61	− 1.24	0.22^a^
Height (cm), mean ± SD	163.91 ± 8.29	162 ± 8.12	1.14	0.26^a^
Weight (kg), mean ± SD	63.57 ± 11.68	60.47 ± 11.13	1.32	0.19^a^
Diagnose				
VHD	36 (78.3%)	34 (69.4%)	0.96	0.33
CAD	10 (21.7%)	15 (30.6%)		
NYHA				
I	2 (4.3%)	0 (0%)	− 1.1	0.27^b^
II	16 (34.8%)	14 (28.6%)		
III	26 (56.5%)	33 (67.3%)		
IV	2 (4.3%)	2 (4.1%)		
Educational level				
None	2 (4.3%)	4 (8.2%)	− 0.5	0.62^b^
Primary	19 (41.3%)	18 (36.7%)		
Junior	14 (30.4%)	19 (38.8%)		
Senior/secondary school	6 (13%)	5 (10.2%)		
College/university	5 (10.9%)	3 (6.1%)		
Marital status				
Married	45 (97.8%)	46 (93.9%)		0.62^c^
Unmarried	1 (2.2%)	3 (6.1%)		
Occupation				
Worker/enterprise/institutional workers	16 (34.8%)	9 (18.4%)	3.35	0.34
Farmer	9 (19.6%)	13 (26.5%)		
Retiree	7 (15.2%)	9 (18.4%)		
Others	14 (30.4%)	18 (36.7%)		
Payment				
Without insurance	5 (10.9%)	4 (8.2%)		0.74^c^
Insurance	41 (89.1%)	45 (91.8%)		
Length of ICU stay (h), mean ± SD	63.72 ± 33.08	66.33 ± 33.70	− 0.38	0.71^a^
Duration of use of corticosteroid (days), median	2 (0.3)	2 (0.2)	− 0.97	0.32^b^
Duration of use of sedatives (days), median	1 (0.2)	0 (0.1)	− 1.1	0.27^b^
Duration of use of analgesics (days), median	0 (0.2)	0 (0.1)	− 1.65	0.1^b^
Duration of MV (h), median	20.42 (16.63, 43.63)	20 (14.63, 42.33)	− 0.71	0.48^b^

Statistical analysis was performed using the chi-square test. *NYHA* Classification of New York Heart Association Heart Function, *VHD* valvular heart disease, *CAD* coronary atherosclerotic heart disease, *MV* mechanical ventilation

^a^Independent *t* test

^b^Mann-Whitney *U* test

^c^Fisher’s exact test

**Table 2:** Comparison between withdrawal and responded patients in length of ICU stay, duration of use of corticosteroid, use of sedative, use of analgesic, and MV hours

Variables	Withdrawal (*n* = 31)	Responded (*n* = 95)	*Z*/*T*	*P*
Length of ICU stay (h)	99.44 ± 64.53	65.07 ± 33.25	2.85^a^	0.01*
Duration of use of corticosteroid (days), median	3 (2.5)	2 (0.3)	− 3.01	0.00*
Duration of use of sedatives (days), median	1 (0.4)	0 (0.2)	− 2.66	0.01*
Duration of use of analgesics (days), median	1 (0.4)	0 (0.2)	− 2.17	0.03*
Duration of MV (h), median	43.5 (19.83, 110.42)	20.17 (16.5, 42.58)	− 2.64	0.01*

Statistical analysis was performed using the Mann-Whitney *U* test, *MV* mechanical ventilation; *P < 0.05

^a^Independent *t* test

### Primary outcome

At 3 months, 83 patients (41 in the intervention group, 42 in the control group) completed the follow-up interview. The number of patients with an IES-R score greater than 35 was 6 of 41 (14.63%) in the intervention group vs 9 of 42 (21.43%) in the control group (risk difference, − 9% [95% CI, − 2% to 21%]; *Z* = 1.65, *P* = 0.10). Both mITT and PP analyses showed there were no significant differences between the two groups in total IES-R scores, symptoms of intrusion, and avoidance scores (*P* > 0.05), while the symptoms of hyperarousal scores were significantly lower in the intervention group than those of the control group (*P* < 0.05) (Table 3).

**Table 3:** Comparison of outcomes between groups post-intervention

Variables	mITT				PP			
	Intervention (*n* = 46)	Control (*n* = 49)	*T*/*Z*	*P*	Intervention (*n* = 41)	Control (*n* = 42)	*T*/*Z*	*P*
Primary outcome								
IES-R total score	20.54 ± 10.93	25.16 ± 11.98	− 1.96	0.052	20.73 ± 12.12	25.17 ± 12.97	− 1.61	0.11
Avoidance score	6.41 ± 4.66	7.80 ± 5.11	− 1.38	0.17	6.71 ± 5.24	7.83 ± 5.53	− 0.95	0.34
Intrusion	9.09 ± 5.32	10.53 ± 4.98	− 1.36	0.18	9.00 ± 5.59	10.48 ± 5.37	− 1.23	0.22
Hyperarousal	5.05 ± 3.52	6.84 ± 3.67	− 2.43	0.02*	5.02 ± 3.73	6.86 ± 3.97	− 2.17	0.03*
Secondary outcomes ICU memories								
Factual memories	7.26 ± 2.52	5.93 ± 2.85	2.14	0.02*	7.26 ± 2.61^c^	5.93 ± 3.09	2.14	0.04*
Feeling memories	2 (1.2)	2 (1.3)	− 1.14	0.26^b^	2 (1.2)^c^	2 (1.4)	− 1.15	0.25^b^
Delusional memories	0 (0, 1.25)	0 (0.1)	− 1.02	0.31^b^	0 (0.2)^c^	0 (0.1)	− 0.77	0.44^b^
PSQI score	8.40 ± 3.11	10.14 ± 4.19	− 2.29	0.02	8.22 ± 3.07	10.19 ± 4.52	− 2.31	0.02*
HADS								
Anxiety score	3 (1.5)	4 (2.6)	− 1.65	0.10^b^	3.10 ± 2.70	4.17 ± 3.00	− 1.70	0.09
Depression score	2.02 ± 2.61	2.98 ± 2.68	− 1.75	0.08	1.78 ± 2.53	3.05 ± 2.87	− 2.13	0.04*
SF-36 total score	120.62 ± 12.09	116.42 ± 15.03	1.50	0.14	120.85 ± 12.65	116.21 ± 16.19	1.45	0.15

Statistical analysis was performed using independent *t* test

*PSQI* Pittsburgh Sleep Quality Index, *HADS* Hospital Anxiety and Depression Scale, *SF-36* the MOS 36-item Short-Form, *mITT* modified intention-to-treat, *PTSD* post-traumatic stress disorder, *PP* per-protocol;*P < 0.05

^a^Chi-squared test

^b^Mann-Whitney *U* test

^c^*n* = 43

### Secondary outcomes

#### Memories of ICU

After 1 month post-ICU, numbers of factual memories were significantly more in the intervention group than those of the control group (*P* = 0.02). However, no significant difference was found in the numbers of memories of feeling and delusional memories. PP analysis results were the same as mITT (Table 3) (comparison pre-intervention in Supplemental Table 1).

#### QoL, anxiety, depression, and sleep quality

After 3 months post-ICU, both mITT and PP analyses showed there were no significant differences between the two groups in total SF-36 scores and HADS-anxiety scores, while PSQI scores were significantly lower in the intervention group than those of the control group (*P* < 0.05). Results of mITT analysis showed no significant difference in HADS-depression scores between groups, while PP analysis found depression scores were significantly lower in the intervention group than those of the control group (*P* = 0.04) (Table 3) (comparison pre-intervention in Supplemental Table 1).

#### Adverse effect

No adverse effect related to ICU dairy was found during the telephone interview 1 and 3 months after discharge from ICU.

## Discussion

These results found that using an ICU diary is not useful for preventing PTSD symptoms, anxiety symptoms, and preserving health-related quality of life in the patients at 3 months post-ICU, while it increases the survivor’s factual memories of ICU at 1 month post-ICU, improves the sleep quality, and prevents the hyperarousal symptom at 3 months post-ICU. The results of this study are not entirely consistent with previous trials [14, 17, 19] and systematic reviews [17, 18]. Similar to a trial published on JAMA, ICU diary is not associated with symptoms of PTSD, symptoms of anxiety, and memories of ICU in ICU survivors [19]. But the results differ from the results of other studies which indicated using ICU diaries reduced symptoms of PTSD [7, 8, 16], symptoms of anxiety, depression [7] post-ICU, and improved health-related QoL during 3-year after ICU [14]. As for the effect on depression symptoms, there may need a large sample and more evidences to test due to the different results by mITT and PP analyses in the present study.

Eleven patients in the intervention group refused to read the ICU diaries, and 11 patients in the control group refused to continue the study and did not like to talk their experiences of the ICU stay. When measured shortly after ICU, a high early prevalence of PTSD symptoms may reflect acute stress disorder (ASD) rather than PTSD [6]. ASD symptoms are similar to the PTSD symptoms that occur within the first month of exposure to a traumatic event [30]. ASD may be triggered by fragmented ICU memories of traumatic or psychotic experiences [16]. These patients who had avoidance behavior such as dislike reading the diary and talking about the ICU experience might have ASD; thus, they may start avoiding any stimulus that reminds them of the traumatic event, such as ICU admission. Reading ICU diary can be stressful, yielding a negative emotional experience for some patients [19], which may result in re-traumatizing the patient. Meanwhile, there were significant differences in the characteristics of patients between responders and non-responders in the study, namely, patients who withdrew from the study had a longer length of ICU stay, duration of use of corticosteroid, use of sedatives, use of analgesics, and duration of mechanical ventilation than those who completed the trial. The use of analgesics [31] and the length of mechanical ventilation [32] were associated with PTSD. All these would imply that the patients with poor mental health were more likely to be those who declined to continue the study, refuse reading the diary, and talk about the ICU experience, leading to deflation of the effect of the diary. We were not able to tell whether they had suffered from ASD in this study. ASD is a risk factor for the development of PTSD [33]. More attention and screening of these patients for ASD symptoms, followed by proper support and treatment, is needed, given the correlation between the two disorders.

In this study, the incidence of PTSD in the control group was 21.43% at 3 months after ICU discharge, which is consistent with the newest result of meta-analysis [6]. Although the incidence of PTSD symptoms was 14.63% in the intervention group, there was no significant difference between groups. There are four possible explanations for the failure of the intervention used in this trial to prevent PTSD symptoms in patients. First, the population was specific to those who underwent selective cardiac surgery, such as heart valve replacement and coronary artery bypass grafting surgery, which was different from patients with an acute critical illness or severe trauma. Second, high risk of attrition bias and small sample size in the study may cause bias when estimating the effect of the diary. Third, the doses of ICU diary intervention and patients’ compliance with the intervention is critical. This study did not measure the number of times the patient reading the diary, which may result in the study being underpowered. Fourth, this was the first trial of ICU diary in patients after ICU discharge in mainland China. Most researches of ICU diary came from western countries and Europe. Any influence on psychological outcomes may have been masked by social and environmental factors such as social-cultural difference, setting, and character traits of the study population.

This study found the use of a diary was useful to fill the patient’s factual memories. As the results of our previous cohort study [34], some patients suffered from delusional memories of ICU at 1 week follow-up. It is known that delusional memories without recall of factual events in the ICU is a predictor of PTSD symptoms, while factual memories, even relatively unpleasant memories for real events during critical illness, may give some protection from anxiety and the development of PTSD symptoms [2]. In this way, reading ICU diaries could help the patients fill their memory gap and reconstruct their ICU stories [15], both for the patients with or without delusional memories of ICU.

Quality of life is a critical, patient-oriented long-term outcome in ICU survivors. This study found the use of ICU diaries did to preserve the quality of life in ICU survivors, which is not similar to those of other studies [14, 17, 18]. Compared with the baseline data, both groups increased the SF-36 scores at post-3 months while without a significant difference between groups post-intervention. It was reported that there was a greater improvement in the quality of life in patients with adult cardiac valve disease at 3, 6, and 12 months after surgical treatment, suggesting surgery treatment is useful to improve quality of life through improving heart function [35]. Therefore, there may indicate a process of self-healing and recovery of quality of life over time after cardiac surgery in our study.

Sleep is a basic need for human beings and recovery after critical illness. It is known that PTSD is characterized by severe sleep disturbances [36]. The significant and new finding in this study is that the use of ICU diary was associated with higher subjective sleep quality during the 3 months after ICU discharge. However, in this study, no matter before surgery and 3 months post-ICU, patients’ mean total scores of PSQI were higher 7 points, suggesting the patients suffered from poor subjective sleep quality. Thus, although in the present study, sleep quality was significantly improved by ICU diaries, this is an area which would benefit from further investigation to enhance this conclusion.

### Limitations of the study

Our study design has a number of limitations, which should be noted. First, the study did not measure patients’ compliance with the intervention, which may cause the study being underpowered when estimating the effect of the diary. Second, high rates of drop-out and small sample sizes were limited to the power of our statistical analyses. Third, the ICU diary was detailed to the patient in the intervention group in the general ward. Thus, activities of nursing care and treatment might interfere with patient involvement in the intervention process and reduce the effectiveness of the intervention. Private room may be required to improve the effect of the intervention. Future study designs should consider including larger samples and multicenters, enhance the doses of intervention, and promote patients’ compliance to enhance the power of the study, then to explore the efficacy and feasibility of ICU diary in China context.

## Conclusions

In summary, our results found that using an ICU diary is not useful for preventing PTSD symptoms, anxiety symptoms, and preserving health-related quality of life in the patients after 3 months of discharge from ICU. But the results clearly demonstrated the use of ICU diaries is useful for increasing the survivor’s factual memories of ICU at 1 month post-ICU, improving subjective sleep quality, and preventing hyperarousal symptoms at 3 months post-ICU. Our pilot study provides a reasonable basis for promoting ICU diary interventions for ICU patients. Adequately powered randomized trials should be developed to provide stronger evidence about the potential beneficial effects of ICU diaries not only on the psychiatric outcomes but also on the quality of sleep and quality of life of patients, as these are likely to be of most value to patients and may benefit from the ICU diary.

## Supplementary information

**Additional file 1 **Supplemental Table 1. Comparison of secondary outcomes between groups pre-intervention. Statistical analysis was performed using Independent t-test; a: Mann-Whitney U test. mITT: modified intention-to-treat; PP:per-protocol; PTSD: post-traumatic stress disorder; PSQI: pittsburgh sleep quality index; SF-36: the MOS 36-item short form; * *P*<*0.05.*

## Data Availability

Not applicable

## Abbreviations

CAD: Coronary atherosclerotic heart disease
CSICU: Cardiac surgery ICU
HADS: Hospital Anxiety and Depression Scale
ICU: Intensive care unit
ICUM: ICU memory
IES-R: Impact of Events Scale-Revised Questionnaire
mITT: Modified intention-to-treat
MV: Mechanical ventilation
NYHA: Classification of New York Heart Association Heart Function
PP: Per-protocol
PSQI: Pittsburgh Sleep Quality Index
PTSD: Post-traumatic stress disorder
QoL: Quality of life
RCTs: Randomized controlled clinical trials
SF-36: Medical outcomes study 36-item Short-Form
VHD: Valvular heart disease
χ^2^: Chi-square
