# Supplementary material for: Effect of an ICU diary on psychiatric disorders, quality of life, and sleep quality among adult cardiac surgical ICU survivors: a randomized controlled trial
Source: Crit Care. 2020 Mar 6;24:81. doi: 10.1186/s13054-020-2797-7 (PMC7060606; doi:10.1186/s13054-020-2797-7)
Supplement: Supplementary file 1 — Additional file 1 Supplemental Table 1. Comparison of secondary outcomes between groups pre-intervention. Statistical analysis was performed using Independent t-test; a: Mann-Whitney U test. mITT: modified intention-to-treat; PP:per-protocol; PTSD: post-traumatic stress disorder; PSQI: pittsburgh sleep quality index; SF-36: the MOS 36-item short form; * P<0.05. [file 13054_2020_2797_MOESM1_ESM.docx]

Supplemental Table 1. Comparison of secondary outcomes between groups pre-intervention

| Variables | mITT | | |  |  | PP | | |  |  |
| --- | --- | --- | --- | --- | --- | --- | --- | --- | --- | --- |
|  | Intervention  (n=46) | Control  (n=49) | T/Z | *P* |  | Intervention  (n=41) | Control  (n=42) | T/Z | *P* |  |
| ICU memory |  |  |  |  |  |  |  |  |  |  |
| Number of factual memories | 6.57±2.40 | 5.71±2.68 | 1.63 | 0.11 |  | 6.59±2.46 | 5.48±2.80 | 1.90 | 0.06 |  |
| Number of feeling memories | 1.89±1.27 | 2.22±1.64 | -1.10 | 0.27 |  | 1.88±1.30 | 2.29±1.66 | -1.25 | 0.22 |  |
| Number of delusional memories | 0 (0,1） | 0 (0,1） | -0.29 | 0.77^a^ |  | 0 (0,1） | 0 (0,1） | -0.15 | 0.88^a^ |  |
| PSQI score | 9.60±4.13 | 10.18±3.83 | -0.72 | 0.47 |  | 9.43±4.13 | 10.55±3.83 | -1.28 | 0.20 |  |
| Anxiety score | 5.72±4.88 | 6.47±4.43 | -0.79 | 0.43 |  | 5.93±4.86 | 6.52±4.48 | -0.58 | 0.56 |  |
| Depression score | 2(0,7) | 4(1,7) | -1.01 | 0.31^a^ |  | 2(0,7) | 3.5(1,7) | -0.74 | 0.46^a^ |  |
| SF-36 total score | 110.29±16.99 | 110.47±16.35 | -0.05 | 0.96 |  | 110.53±17.67 | 110.28±16.95 | 0.07 | 0.95 |  |

Statistical analysis was performed using Independent t-test; a: Mann-Whitney U test. mITT: modified intention-to-treat; PP:per-protocol;

PTSD: post-traumatic stress disorder; PSQI: pittsburgh sleep quality index; SF-36: the MOS 36-item short form; * *P＜0.05*
